# Maternal health service utilization from urban health extension professionals and associated factors among women who gave birth in the last one year in Ambo town, Oromia regional state, Ethiopia, 2018

**DOI:** 10.1186/s12889-020-08641-5

**Published:** 2020-04-15

**Authors:** Kebede Megerssa Berri, Yeshi Kumera Adaba, Teka Girma Tarefasa, Nagasa Dida Bededa, Daniel Belema Fekene

**Affiliations:** 1 Ambo town health office CDC department expert, Ambo, Ethiopia; 2 Department of Child Health, Ambo University College of Medicine and Health Sciences, Ambo, Ethiopia; 3 Department of public health, Ambo University College of Medicine and Health Sciences, Ambo, Ethiopia; 4 Department of Midwifery, Ambo University College of Medicine and Health Sciences, Ambo, Ethiopia

**Keywords:** Maternal health services utilization, Urban health extension professional, Urban health extension program

## Abstract

**Background:**

Despite the existence of urban and rural health extension workers maternal mortality and morbidity remain, as a public health problem in Ethiopia. The utilization of maternal health services from UHE-ps is key activities, which contribute to the reduction of maternal morbidity and mortality. This study aimed to assess maternal health service utilization from urban health extension professionals and associated factors among women who gave birth in the last one year in Ambo town.

**Methods:**

Community-based cross-sectional study conducted from February to March 2018 among women who gave birth in the last year before the study period, in Ambo town, Oromia, Ethiopia. The Data collections were through face-to-face interview, the Focus discussion group was done using a semi-structured questionnaire. Descriptive, bivariate and multiple logistic regressions computed by SPSS version 20. Statistical significance was considered at *p* < 0.05 and the strength of statistical association was assessed by odds ratio with 95% confidence intervals.

**Result:**

From the total respondents, only 57(14.2%) women utilized maternal health services from urban health extension professionals during their recent birth. Regarding maternal health services utilization from urban health extension professional’s ANC, Delivery and PNC were 159 (39.7%), 115 (28.7%) and 76 (19%) respectively. The variables, such as graduated as model family (AOR: 2.4; 95% C I: 1.20, 4.78), home visit during pregnancy within month (AOR: 11.6; 95% CI: 3.60, 37.17), awareness on pregnancy danger sign (AOR: 3.8; 95% CI: 1.62, 8.92) and parity (AOR: 2.8; 95% CI: 1.06, 7.61) were factors affecting maternal health services utilization from urban health extension professionals.

**Conclusion:**

The utilization of maternal health services from urban health extension professionals was found to be low. Being graduated as a model family, awareness on pregnancy danger sign, parity and urban health extension professional’s home visit during pregnancy had a positive statistically significant association with maternal health services utilization from urban health extension professionals. Therefore, considering the identified associated variables could increase and sustain maternal health services utilization from urban health extension professionals.

## Background

Maternal health care utilization is the care given for women form pregnancy up to the postpartum period. It includes ANC (ante natal care), delivery care, and PNC (post natal care) services. This utilization of care has been recognized as great importance since the satisfaction of the basic needs of the baby at every phase of their life is closely linked to the well-being of the mother [1, 2].

The use of ANC, skilled delivery attendants and PNC are recognized as key maternal health care to improve health of women and children [3]. The health extension program is an innovative community-based initiative, which services as the primary vehicle for the implementation of community-centered essential health care packages. The program is aimed to create a healthy environment and healthful living by making available essential health services at the grassroots level by improving the health-seeking behavior of the urban community through comprehensive, organized, economically and technically feasible health interventions [4, 5].

The government of Ethiopia launched an innovative program called Urban Health Extension Program (UHEP) in 2009, intended to ensuring health equity by creating demand for fundamental health services through the provision of health information at the household level, school, and youth centers and improving access to health services through referral to health facilities [3].UHP expected to provide 15 programs. The services are grouped into four main themes: hygiene and environmental sanitation, family health care, prevention and control of communicable and non-communicable diseases, and injury prevention, control, first aid, referral and linkages [5, 6].

The program has been implementing in three settings of different socioeconomic, cultural, and environmental conditions. The UHE-ps are expected to visit households 3 days per week in their catchment from this the 2 days visit are focus on households of a pregnant mother and those who have under 5-year children [7].

Globally, maternal health target of reducing maternal mortality ratio (MMR) by MDG is ¾ but only dropped by nearly 38% over the past 25 years, According to the latest United Nations (UN) estimate maternal mortality rate was 462 /100000 live births versus 11/100000 live births in high-income countries respectivel y[8–10].

Approximately, 94% of all maternal deaths occur in low and lower-middle-income countries.Sub-Saharan Africa accounts 196,000 and Southern Asia 58,000 of maternal deaths respectively [8].

In Ethiopia, According to EDHS 2016, the estimate of the MMR dropped to 412 /100,000 live births; reduce by 69% in the last 25 years. Although Ethiopia is one of the countries that have recently reported improvement in reducing maternal mortality using low-cost impact interventions using health extension program that provides basic health care services, Still data from EDHS 2016 shows that estimated.

The maternal mortality rate is higher than estimated MMR of developing countries which is about 239 /100,000 live births [10–12].

Ethiopian Federal Ministry of Health has applied a multi-pronged approach to reduce maternal and newborn morbidity and mortality by improving access and equity service strengthening community-based and facility-based maternal and newborn care services [10, 13].

Previous studies mainly focused on assessing utilization of the urban health extension program rather than maternal health care from UHE-ps throughout the pregnancy up to postnatal period [14–16]. Further, understanding in detail factors affecting maternal health care and its utilization from UHE-ps is crucial. Considering the scarcity of reliable and documented evidence on maternal health service utilization from UHE-ps in the study area, the aimed of this study is to identify maternal health service utilization from urban health extension program which will planning and implementing programs towards utilization of this service, which has a great role for reduction of maternal morbidity and mortality.

## Methods

### Study area and period

The study conducted from February to March 2018 in the Ambo town West Showa zone, Oromia, which was located 114 km to the west of Addis Ababa, the capital city of Ethiopia. In the town, there are one General Hospital, one Referral Hospital, 2 government health centers and twenty-seven private clinics providing health services to the community [17]. According the national population and housing census, the total population of the town for 2018 was 83,053; Men constitute 41,692 (50.2%) and Women constitute 41,361 (49.8%). The estimated pregnant mother was in 2882. From this under one year Children were **2666**, Women of childbearing age (15–49) **18,379;** total House Hold accounts about **17,303 (**17).

The town divided into six administrative Keble. The three Keble in the town started implementing the urban health extension program since 2009. The population is obtaining community health services from 21 UHE-ps and 7 HEWs provide preventive services to an average of 617 households per UHE-ps and HEWs [17, 18].

All women in Ambo town who gave birth where source population and all women who gave birth in the last year, who lives in the town for more than six months, were included. On the contrary, those who were very sick were not included in the study.

### Study design

A community-based cross-sectional study that employed both qualitative and quantitative data collection methods was carried out to assess factors utilization of maternal health care services from UHE-ps.

### Sample size determination and sampling technique

The sample size was calculated by single population proportions formula with the assumptions of P is the maternal health service utilization from UHE-ps *P* = 50%, with a 95% confidence level. By considering a 10% non-response rate, the final sample size was 403. The three Keble implementing urban Health extension program from 2009 was selected from the total six kebeles in the town by lottery method, from those selected kebeles, households whose mothers who gave birth in the last one year were live in were selected randomly from the sampling frame obtained from kebele health office and health extension workers. The sample size for each kebeles was determined proportionally to the number of mothers who gave birth in the last year within each kebeles.

For qualitative, focus group discussions (FGDs) and semi-structured open-ended (FGD) guide was used to triangulate responses obtained by the structured questionnaire on the maternal health care utilization from UHE-ps (specifically ANC, delivery and postnatal care and associated factors).Non-probabilistic purposive sampling technique was used. Each FGD consisted of eight women selected by Health developmental army (HDA) from the three kebele from women who gave birth last year not participated in a quantitative study.

### Variables

#### Dependent variable

Utilization of maternal health service from UHE-ps: ANC, delivery related service and PNC services.

#### Independent variables

**Socio-demographic characteristics**: age, marital status, educational status of mother.

Occupation, ethnicity, religion, family income and partner education.

**Past Obstetric History:** No of pregnancy, Place of delivery for index child, Pregnancy type (planned/unplanned.

**Urban Health professional’s utilization:** UHE-ps frequency of home visit, UHE-ps availability in catchment area, social interaction of UHE-ps with mother and UHE-ps activities during home visit.

**Maternal Health program:** awareness on maternal health service, awareness on pregnancy danger sign and graduated model family.

#### Operational definition

**ANC Utilization from UHE-ps:** women who get at least one ANC service or referred to health facility for ANC service from UHE-ps during the last pregnancy (14).

**PNC utilization from UHE-ps:** women who get at least one PNC service or referred to Health facility for PNC services by UHE-ps within 42 days after of the respondent’s recent birth [14].

**Delivery Service Utilization from UHE-ps:** Considered when UHE-ps gave at least one of the following delivery related care during the respondent recent birth like; identification of complication during labor, giving ambulance number and referred to health facility, identification of sign of labor and referred to health facilities.

**Urban Health extension professional**: Diploma nurse who trained on 15-health extension program for additional three months.

**Graduated Model Household:** defined as households that attend the training given by UHE-ps on 15 program for 60 h and implement at least 75% of UHEP.

**Utilization of maternal health service from UHE-ps** was categorized into users and non-users. Users were women who have received at least once for each of the care consecutively from **UHE-ps** and non-users were mothers who didn’t received at least once for each care consecutively from **UHE-ps.**

**Utilized maternal health service from UHE-ps**.**:** is utilization of at least one ANC service, delivery related care and at least one PNC within 42 days of delivery from UHE-ps during the respondent’s recent childbirth [14].

#### Data collection tool/process

Data was collected through face to face interview. The questionnaire has different parts and the tool adapted from previous literature in different parts of the world and modified according to the local context [4, 12]. Six diploma nurses were recruited as data collectors and BSC nurses as supervisors. Besides, the data collectors were trained for one day on the purpose of the study, interviewing technique and issue on confidentiality before the start of data collection. During the data collection, regular supportive supervision and discussion with data collectors and supervisors were done. Every day, the supervisors have checked all the filled questionnaires for completion and clarity.

The FGD guides prepared for each target participant in the qualitative study. Audio recorder and note taking did for qualitative data in FGD with the permission of discussants.

### Data analysis

The coded data were entered into EPI data version 3.1 and exported to SPSS version 20 for analysis .descriptive statistics (mean standard deviations and percentages)were computed to present the data and describe the study participant. Bivarate analysis was undertaken to assess the association between all independent variables and the outcome variable. Of these, variable with *p*-value <_0.25 in the bivariate analysis were taken and analyzed by the backward stepwise method in multiple logistic regression models. Adjusted odds ratios with their respective 95% CI were computed and variables, with p-value < 0.05 were taken as a statistically significant associated with of maternal health service utilization from urban health extension.

The qualitative part note taking and tape recorder interview was done in the local language and it was translated into English. The data was organized and analyzed to the major thematic areas, and then the result was supported by qualitative data to supplement the quantitative finding for its consistency.

### Data quality control

The data collection tool was prepared English and translated into local language Afan Oromo by the translator, and then translated back to English by a third person to check for consistency and accuracy. Training was given to both the data collectors and supervisors for one day on the purpose of the study, data collection tools, and procedure, how to interview, maintaining confidentiality and privacy. A pretest was done on 5% of the total study participant at Gudar town two weeks before the commencement of data collection and necessary adjustment was made.

### Ethical and legal consideration

Ethical clearance obtained from Ambo University; College of Medicine &Health Science ethical review committee. A supportive letter obtained from Ambo University College of Medicine &Health Science Post Graduate Office to Ambo town Health Office. The supportive letter was written from the Ambo town health office to kebele administrative and health centers. Permission obtained from the Keble administrative and health centers before data collection. Informed verbal consent obtained from the study participants. Participants were informed that participation on a voluntary basis and they can withdraw at any time if they are not comfortable about the questionnaire. Personal identity was not included in the written questionnaires to ensure participants’ confidentiality.

## Results

### Socio-demographic characteristics

In this study, a total of 401 participants had fully responded to the questionnaire making a response rate of 99.5%. The mean age of the respondents was 27.18 with a standard deviation of 5.13 years. The majority of respondents were Oromo 383(95.5%) by ethnicity, Protestant 201(50.1%) by religion. Concerning their occupation, about 274(68.3%) was a housewife and whereas, 358 (89.3%) respondents are currently married. Of the total respondents, 153 (38.2%) were attended primary school (Grade 1–8) and 38 (9.5%) of them have no have formal education. The majority, 142 (35.4%) of participants have 1651–3200 Ethiopian birr monthly household income with a mean of 3308.03(SD = + 2396.52). Regarding partner occupation, 168 (46.9%) of them are currently employed. Whereas partners educational status 164 (45.8%) joined college and 21 (5.9%) are not attended formal education (Table 1).

**Table 1:** Socio-Demographic characteristics of mothers, who gave birth in the last 1 year, Ambo town, Oromia, Ethiopia, 2018(*n* = 401)

Variables	frequency	percentage
Age categories		
18–24 age	112	27.9
25–34 age	245	61.1
>=35 age	44	11
Religion		
Orthodox	167	41.6
Muslim	11	2.7
Catholic	18	4.5
Protestant	201	50.1
Wekefata	4	1
Ethnicity		
Amhara	17	4.2
Tigre	1	0.2
Oromo	383	95.5
Mother’s occupation		
House wife	274	68.3
merchants	46	11.5
Employed	81	20.2
Husbands Occupation		
Farmers	26	7.3
Un employed	22	6.1
Merchants	49	13.7
Daily labor	93	26
Employed	168	46.9
Husband’s Educational status		
no formal education	21	5.9
Grade 1–8	91	25.4
Grade 9–12	82	22.9
College and above	164	45.8
no formal education	38	9.5
Grade 1–8	153	38.2
Grade 9–12	97	24.2
College and above	113	28.2
monthly income		
<=1650	111	27.7
1651–3200	142	35.4
3201–5250	85	21.2
>=5251	63	15.7

### Urban health extension professional’s utilization related factors

The study shows that 307 (76.6%) of respondents had awareness of Urban Health extension professionals (UHE-ps) available in their jobs. From those respondents that had awareness, 44 (14.3%) of them were get home visit on monthly or weekly base form UHE-ps. For instance, a 28-year-old mother who gave birth last year and a member of the Health Development Army said that*“…Truly speaking, urban health extension professionals have visited me every three months when I was pregnant and after I gave birth”.* Moreover, about 264 (86%) of respondents were educated on the Urban Health extension program (UHEP) at households level during a home visit and the rest 43 (14%) were educated and demonstrated on UHEPs. The above quantitative results supported by qualitative findings. *“UHE-ps thought me the benefit of institutional delivery and encouraged me to give birth in a health facility. She also followed me even after delivery and advised me of exclusive breastfeeding and personal hygiene practice.”* (**28-year-old mother member of Health Development Army-FGD).**

**Fig. 1:**
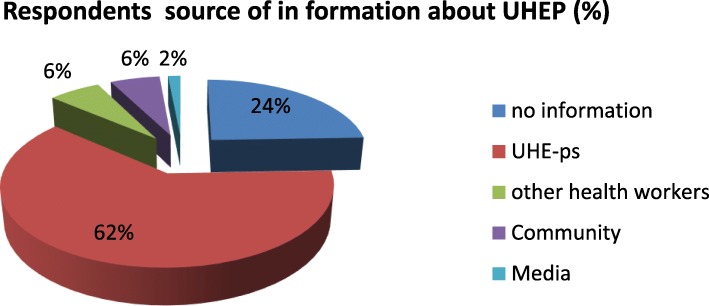
Respondents source of information about UHEP Ambo town, 2018 (*n* = 401)

From 307 study respondents, 49 (16.3%) responded that the social interaction of UHE-ps was very good the others 130 (42.3%), 93 (30.3%) and 30(10%) were responded as good, fair and poor respectively and the rest three (1%) were very poor. The study also shows that respondent’s source of information on UHEPs was mainly UHE-ps (62%) and the others source of information was other health personnel and Community each (6%), the rest 74(24%) of them were no information on UHEPs (Fig. 1). Concerning model family graduation 97(24.2%) of the respondents were graduated as a model family whereas 304(75.8%) was no graduated. *“…we (UHE-ps) work closely as a Family with the women when we go to their home; they also accept us their friends”. (****A 30-year Ambo Town UHE-ps –FGD)****“The one member of the FGDs indicated; the UHE-ps were educating us on UHEP using different opportunities like pregnant women conference, during the home visit and at community meetings. Therefore, we are conscious of UHEP.***(30 year -women from health developmental army -FGD).**

*“The mother discusses with us about their health-related issues during their pregnancy and after delivery in a friendly manner. They have confident and happy with the services we provide for them.” (A****30-year UHE-ps –FGD).***

### Maternal health service utilization

**Fig. 2:**
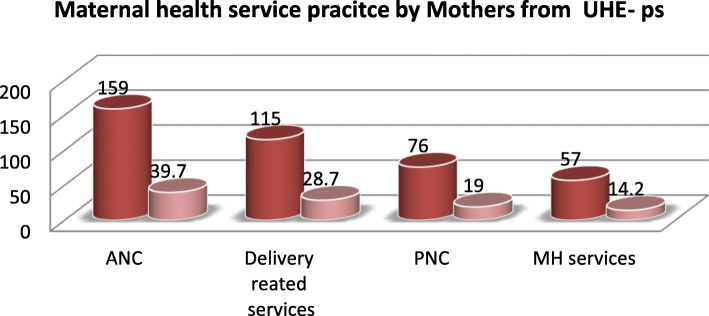
maternal health service utilization by respondent from UHE-ps in Ambo town, 2018(*n* = 401)

The study revealed that out of 401 respondents, who utilized maternal health service starting from pregnancy to postnatal period from urban health extension professionals during their most recent childbirth were 14.2% (Fig. 2).

The eight participants of FGDs from UHE-ps expressed that they educate the mothers as well as the community for the last eight years on health-seeking behavior especially on benefits of ANC, institutional delivery and PNC. Therefore, the majority of the respondents showed that the need for using Maternal health care just during their pregnancy and puerperium period from health professionals. Few of the respondents show dalliance of seeking services from UHE-ps. From this, we have to do more to break these challenges the health center also should prepare them to do more. ***(Ambo town UHE-ps-FGD).***

*“As I think even though we couldn’t reach every household as schedule the mothers have awareness on maternal health they would receive maternal health services from health facility through self-referral, therefore, all would receive maternal health services indirectly even though we didn’t provide them directly”.****(Ambo town UHE-ps-FGD)***

### Antenatal care services

Among those 401 respondents, 164(40.9%) were visited by UHE-ps during their last pregnancy 159(39.7%) of them received Antenatal care from urban health extension professionals during their most recent pregnancy. From those mothers who get ANC from UHE-ps more than two-third of 106 (68%) respondents were attended less than four Antenatal care in their most recent pregnancy and 50 (12.5%) respondents were attended four and above ANC services during the most recent pregnancy from UHE-ps. Moreover 112(27.9%) of pregnant women provided referral paper for ANC services to Health institution, 101 (25.2%) provided health education on ANC and 20 (5%) of them provided health education on pregnancy danger sign by UHE-ps.

One hundred eighteen (74.2%) women engaged ANC at the time of the second trimester, 35 (22.2%) were attended ANC at their first trimester and the rest 6 (3.8%) engaged ANC services at third trimester by urban Health extension professionals. The respondents were asked about awareness on pregnancy dangers sign, from the total study 259 (64.6%) of them were aware of pregnancy danger sign and the rest 142 (35.4%) were not aware of it. When we see each, 141 (35%) of them were aware of Headache, and the least 16(4%) conscious on the retained placenta.

*The majority of the UHE-ps in FGD participants reported that for the last eight to nine years they were teaching the community on pregnancy danger signs not only the mother but also other members of the community. Therefore, they believe that every mother has awareness of Pregnancy danger sign.****(Ambo town UHE-ps - FGD).***

*Member of UHE-ps FGD said that; “According to my Catchment area, I taught about UHEPs almost all in a different setting not only maternal health services but also all UHEP packages especially during Model family training. Concerning maternal health services we are conducting pregnant mothers conference at Kebele together with health center Mid-wafers nurse this helps the mothers to seek health center services during their pregnancy period”.****(UHE-ps- FDG)***

### Delivery services utilization

Among the respondents 387 (96.5%) of their recent births were at health institutions; like Hospital, Health Center and Private Health Facility 169 (42.1%), 214 (53.4%) and four (1.0%) respectively. One discussant from FGD said; *“In our kebele, each pregnant woman gave delivery in health institution because every one of us was informed by UHE-ps during pregnant women conference”.****(28 year- women from HAD –FGD).***

From those study participants, 115 (28.7%) were received delivery-related services from UHE-ps. Those services like; 42 (10.5%) of them were identified complication during labor and referred to Health facility, 36 (9%) of them were identify the sign of labor and referred to the health facility and 41 (10.5%) of them were given ambulance number and referred to the health facility. Regarding decision-making power towards selecting the place of delivery 206 (51.4%) of respondents decide together with their partners, 178 (44.4%) of respondents were decide by themselves and 16(4%) of the respondent’s place of delivery were decide by their partners.

All UHE-ps participated in FGD described that UHE-ps educate the pregnant mothers about the benefit of institutional delivery, give them referral paper and we provide Ambulance phone numbers. For example, one discussant from UHE-ps said; *“during the home visit, I ask for the presence of a pregnant mother or not if pregnant mother was there I asked for the month of pregnancy if the mother was at term I referred her to a health facility for delivery services”. (****Ambo Town-UHE-Ps-FGD)***

### PNC services utilization

The overall postnatal care service utilization of the respondents from Urban Health extension professionals were 76(19%). When we see separately the study shows 10(2.5%) of them were received within 24 h of postpartum period, about 18(3.5%) within 2–3 days after delivery, 13(3.2%) within 4–6 days of delivery and the others 39 (9.7%) were received PNC services after 7 days. Regarding PNC follow up, 52 (13%) utilized PNC care only 1 time and 11(2.7%) received PNC for three or more times.

For example, one member of FGD from the women’s developmental army who gave birth in the last one year said; *“UHE-ps visited me within six weeks after I gave childbirth and she (UHE-ps) asked me about discharge status since delivery and counseled me on exclusive breastfeeding”.***(25-year-FGD-female discussant).**

### Factors independently associated with utilization of maternal health service from UHE-ps

The study showed that variables such as graduated model family, awareness on pregnancy danger sign, frequency of home visit by UHE-ps, social interaction of UHE-ps and Parity were found to be significantly associated with maternal health service utilization from UHE-ps.

In a multivariate model analysis, graduated model family (AOR = 2.399, 95% CI (1.203–4.784)), awareness on pregnancy danger sign (AOR = 3.798, 95% CI (1.618–8.922)), frequency of home visit by UHE-ps (AOR = 11.569, 95% CI (3.601–37.172)) and Parity (AOR = 3.230, 95% CI (1.237–8.434) were the variables that showed significantly associated with Maternal Health Services utilization from UHE-ps.

The study revealed that women of Ambo town, who graduated as a model family were 2.4 times more likely utilizing maternal health services from urban health extension professionals than women who had not graduated as a model family (AOR = 2.399, 95% CI (1.203–4.784)).

Respondents who visited frequently in monthly or weekly were utilized maternal health service from UHE-ps 11.6 times when compared with those women visited every nine-month or latter(AOR = 11.569, 95% CI (3.601–37.172)). Women who had awareness of pregnancy danger signs were utilized maternal health services from UHE-ps 3.8 times more likely than women who had no awareness of pregnancy danger sign (AOR = 3.798, 95% CI(1.618–8.922)). The odds of utilizing Maternal health services from UHE-ps among women of primiparas were 2.8 times more than women of parity four and above (AOR = 2.834, 95% CI (1.056–7.605)). Whereas women who had parity of two were 3.2 times utilize Maternal health services more likely than women of parity four and above do (AOR = 3.230, 95% CI (1.618–8.922)Table 2).

**Table 2:** Bivariate and multivariate logistic regression analysis of factors independently associated with utilization of maternal health service from UHE-ps, Ambo town, Oromia, Ethiopia, 2018

Variable	Category	Maternal health service Utilization from UHE-Ps		COR (95%CI)	AOR (95%CI)
		Yes: n (%)	No: n (%)		
Graduated as model family	Yes	24(24.7%	73(75.3%)	2.7(1.50–4.85)*	2.4(1.20–4.78)*
	No	33(10.9%)	271(89.1%)	1	1
Frequency of home visit	Monthly or weekly	17(38.6%)	27(61.4%)	9.3(3.13–27.72)*	11.6(3.60–37.17)*
	Quarterly	26(24.1%)	82(75.9%)	4.7(1.71–12.85)*	5.5(1.90–15.85)*
	Bi-annually	9(11.8%)	67(88.2%)	1.9(.634–6.23)	2.1(.65–6.98)
	Every nine month	5(6.5%)	250(81.4%)	1	1
Awareness on Pregnancy danger sign	Yes	49(18.9%)	210(81.1%)	3.9(1.79–8.510)	3.8(1.62–8.92)*
	No	8(5.6%	134(94.4%)	1	1
Parity	one delivery	18(14.5%)	106(85.5%)	1.9(.79–4.60)	2.8(1.06–7.61)*
	two delivery	19(19%)	81(81%)	2.6(1.09–6.36)*	3.2(1.24–8.43)*
	three delivery	12(15.2%)	67(84.8%)	2(0.78–5.20)	1.8(.64–4.87)
	> = 4 delivery	8(8.2%)	90(91.8%)	1	1

*= *P* < 0.05 Cut off point for ‘multiple logistic regression

## Discussion

The study revealed that ANC services utilization delivery service and PNC utilization, from urban health extension, were 39.7, 28.7, and 19% respectively. This finding is higher than the study done in Abuna Gindeberet, West Shoa Zone which showed that maternal health care service utilization from health extension workers like Antenatal care was 4.7%, delivery service 2.9% and postnatal care 4.5% among the study participants [4]. Also higher than results done in Tigray 22.5% of the mothers used ANC, delivery by HEW 1.1% and PNC from HEW was 3.4% [19]. The observed difference might be due to the previous studies were done in both urban and rural areas where as; this study was done only in an urban area. However, it is lower than the findings from south and north central ANC 41.6% and PNC 57.9% [20]. The observed difference might be due to Socio-demographic deference and the study done in two regions that were mentored by Addis Ababa University might increase the utilization of the above services.

Being a graduated model family is the factors affecting maternal health services utilization from UHE-ps. women who were graduated as a model family were 2.4 times more likely to utilize maternal health service from UHE-ps as compared to those who were not graduated as a model family (Adjusted Odds Ratio (AOR): 2.4; 95% CI: 1.20, 4.78). This is supported by the studies conducted in Tigray, North Ethiopian, Bishoftu town and Abuna Gindeberet [4, 19, 21]. This might be due to households’ have to go through several steps to graduate as a model family and effective transfer of health knowledge through the urban health extensions professionals to their model households. Moreover, it might be due to good psychological acceptance of the program packages among the participants rather than those who did not participate and utilize the packages.

This study also indicated that having awareness of pregnancy danger signs is significantly associated with the utilization of maternal health services from urban health extension professionals. Those mothers’ women who had awareness of pregnancy danger signs were 3.8 times more likely to utilize maternal health services from UHE-ps than women who had no awareness of pregnancy danger signs (AOR): 3.8; 95% CI: 1.62,8.92). This is supported by the studies conducted in Boricha district, and Kafa zone, Southwest [22, 23]. This might be due to knowing their danger and the consequence can make the women seeking information from health urban health extension professionals. *This was also triangulated by the qualitative component as* teaching the community on pregnancy danger signs not only the mother but also other members of the community to have awareness of Pregnancy danger sign.

Mothers who visited by UHE-ps on monthly or weekly were 11.6 times more likely utilized maternal health services from UHE-ps compared with mothers visit every nine-month or beyond that (AOR):11.6; 95% CI: 3.60, 37.17) and mothers visited by UHE-ps every quarterly utilize 5.5 times when compared with those mothers who visited beyond nine-month (AOR): 5.5; 95% CI: 1.90, 15.85). This finding was agreed with the study conducted in Jimma Zone, north and south-central Ethiopia, [20, 24]. *This was also triangulated by the qualitative component as home visit within appropriate time to utilize the package early as possible* i.e. *exclusive breastfeeding* This might be continuous support of urban health extension professionals can increase the potential to utilize health facilities.

Parity was significantly associated with the utilization of maternal health services from UHE-ps in this study. Women of primiparas 2.8 times (AOR): 2.8; 95% CI: 1.06, 7.61) and women whose parity of two were also 3.2times (AOR): 3.2, 95% CI: 1.24–8.43) more likely utilize maternal health services UHE-ps compared with parity of four and above. Study from Rwanda, Kafa zone, Southwest and Holeta town in line with this study [23–26]. This is might be duet o women experiencing first births and high order births are used as clinical markers for identifying individual women who need a referral or extra care in pregnancy.

### Limitation of the study

There could be recall bias since the women asked for events within the last twelve months before the study. The cross-sectional nature of the study has inherent limitations for establishing cause and effect relationships. There are limited tools related to the research topics. As the study was carried out in Ambo town three Keble, the findings may not be generalized to the whole country.

## Conclusion

This study found the utilization of maternal health service from urban health extension Professionals was only 14.2%. Awareness on pregnancy danger sign, frequency of home visit by urban health extension professionals, number of parity and model family graduation are factors that are significantly associated with utilization of maternal health service from urban health extension professionals. Therefore, encourage model family training, conduct a regular home visit and educate women on pregnancy danger signs and health-seeking behavior are important.

## Data Availability

Full data for this research is available through the corresponding author up on request.

## Abbreviation

AOR: Adjusted Odd Ratio
ANC: Antenatal Care
COR: Crude Odd Ratio
EDHS: Ethiopian Demographic and Health Survey
FGD: Focus Group Discussion
MHC: Maternal Health Care
MMR: Maternal Mortality Ratio
UHE-ps: Urban Health Extension Professionals
WHO: World Health Organization

## References

[1] Tabassum Firoz, Doris Chou, Peter von Dadelszen, Priya Agrawal, Rachel Vanderkruik, Ozge Tunçalp et al. Measuring maternal health: Focus on maternal morbidity. Bull World Health Organ 2013. 2013;91(794–796). 10.2471/BLT.13.117564 PMC3791656 24115804

[2] WHO (2017). Maternal health.

[3] Tarekegn (2014). Determinants of maternal health service utilization in Ethiopia: analysis of the 2011 Ethiopian demographic and health survey. BMC Pregnancy and Childbirth.

[4] Kelbessa Z, Baraki N, Egata G (2014). Level of health extension service utilization and associated factors among community in Abuna Gindeberet District, west Shoa zone, Oromia regional state, Ethiopia. BMC Health Serv Res.

[5] Sibamo EL, Berheto TM (2015). Community satisfaction with the urban health extension service in South Ethiopia and associated factors. BMC Health Serv Res.

[6] Koblinsky M, Frances T, Gaym A, Karim A, Carnell M, Tesfaye S (2010). Responding to the maternal health care challenge: The Ethiopian Health Extension Program. Ethiop J Health Dev.

[7] FMOH. Reproductive, maternal, New born and Child Health,Urban Health Extension Program Integrated Refresher Training,2017.

[8] Trends in maternal mortality (2019). 2000 to 2017: estimates by WHO, UNICEF, UNFPA, World Bank Group and the United Nations Population Division.

[9] Alkema L, Chou D, Hogan D, Zhang S, Moller A-B, Gemmill A, Fat DM, Boerma T, Temmerman M, Mathers C, et al. Global, regional, and national levels and trends in maternal mortality between 1990 and 2015, with scenario-based projections to 2030: a systematic analysis by the UN maternal mortality estimation inter-agency group. Lancet. 2016; 387(10017):462–474. 1. 10.1016/S0140-6736(15)00838-7 PMC5515236 26584737

[10] Kok Maryse C, Kea Aschenaki Z, Datiko Daniel G, Broerse Jacqueline EW, Marjolein D, Miriam T (2015). A qualitative assessment of health extension workers’ relationships with the community and health sector in Ethiopia: opportunities for enhancing maternal health performance. Hum Resour Health.

[11] WHO. Assessing Progress in Africa toward the Millennium Development Goals). 2014.

[12] Central Statistical Agency. Ethiopia Demographic and Health Survey 2016: Key indicators report. Addis Ababa, Ethiopia: 2016.

[13] Dutamo Z, Assefa N, Egata G (2015). Maternal health care use among married women in Hossaina,Ethiopia. BMC Health Serv Res.

[14] Tafesse N, Gesessew A, Kidane E (2019). Urban health extension program model housing and household visits improved the utilization of health Services in Urban Ethiopia: a community-based cross-sectional study. BMC Health Serv Res.

[15] Yitayal M, Berhane Y, Worku A (2014). Health extension program factors, frequency of household visits and being model households, improved utilization of basic health services in Ethiopia. BMC Health Serv Res.

[16] Girmay AM, Evans MR, Gari SR, Gebremariam AG, Reta MT (2019). Urban health extension service utilization and associated factors in the community of Gullele sub-city administration, Addis Ababa, Ethiopia. Int J Community Med Public Health.

[17] THO Ambo (2017). Ambo town health office profile.

[18] THO Ambo (2017). Urban Health extension program base line data.

[19] Kassyou H. Factors affecting antenatal care attendance in Maichew town. Southern Tigray, Degree of Master of Science in Population Studies edn, Institute of Population Studies College of Development Studies, Addis Ababa. 2008.

[20] Fantahun AM, Kesteberhan A, Alemayehu M, Seifu H, Meselech A, Saifuddin A (2014). Effect of an innovative community based health program on maternal health service utilization in north and south central Ethiopia: a community based cross sectional study. Reprod Health.

[21] Gebreegziabher EA, Astawesegn FH, Anjulo AA (2017). Urban Health Extension Services Utilization in Bishoftu Town, Oromia Regional State,Central Ethiopia. BMC Health Serv Res.

[22] Wakgari N (2017). Antenatal care utilization and its associated factors among pregnant women in Boricha District, southern Ethiopia. Diversity and Equality in Health and Care.

[23] Negalign Berhanu Bayou, Yohannes Haile Michael Gacho. Utilization of Clean and Safe Delivery Service Package of Health Services Extension Program and Associated Factors in Rural Kebeles of Kafa Zone,South West Ethiopia. Ethiop J Health Sci. 2013 July 2013;Vol. 23( No. 2). PMC3742885 23950624

[24] Birhanu (2013). Mothers’ experiences and satisfactions with health extension program in Jimma zone, Ethiopia: a cross sectional study. BMC Health Serv Res.

[25] Kidist B, Yohannes D, Desalegn W (2013). Determinants of maternal health care utilization in Holeta town, Central Ethiopia. BMC Health Serv Res.

[26] Rurangirwa (2017). Determinants of poor utilization of antenatal care services among recently delivered women in Rwanda; a population based study. BMC Pregnancy and Childbirth.

